# HRS-4642 combined with nimotuzumab in the treatment of recurrent or metastatic pancreatic ductal adenocarcinoma: study protocol of a single-arm, prospective phase Ib/II trial

**DOI:** 10.3389/fphar.2025.1562481

**Published:** 2025-07-04

**Authors:** Qingqing Leng, Jitao Zhou, Xin Wang, Pei Zhang, Huanji Xu, Dan Cao

**Affiliations:** Division of Abdominal Tumor Multimodality Treatment, Cancer Center, West China Hospital, Sichuan University, Chengdu, Sichuan, China

**Keywords:** pancreatic ductal adenocarcinoma, KRAS G12D mutation, HRS-4642, nimotuzumab, combination therapy

## Abstract

**Background:**

Pancreatic tumors are highly lethal and a leading cause of cancer mortality. While systemic chemotherapy is the mainstay for advanced disease, its efficacy remains limited. KRAS mutations occur in approximately 88% of pancreatic ductal adenocarcinoma (PDAC), of which KRAS G12D comprises up to 39.5%. Despite the promise of KRAS G12D inhibitors, drug resistance persists. Combining KRAS and EGFR inhibitors has shown clinical efficacy in select solid tumors.

**Objectives:**

To assess the safety and efficacy of HRS-4642 (KRAS G12D inhibitor) plus nimotuzumab (EGFR inhibitor) in patients harboring the KRAS G12D mutation, recurrent/metastatic PDAC refractory to standard systemic therapy.

**Design:**

This is an open-label, single-center, exploratory clinical trial.

**Methods:**

This study will enroll patients with histologically or cytologically confirmed recurrent or metastatic PDAC harboring the KRAS G12D mutation, who have documented disease progression or intolerance to first-line systemic therapy. In Phase Ib, the safety profile of the investigational agent HRS-4642 will be assessed starting at an initial dose of 1,200 mg administered every 2 weeks (Q2W). Dose reductions to 1,000 mg Q2W or 800 mg Q2W will be implemented for dose-limiting toxicities. The recommended Phase II dose (RP2D) of HRS-4642 in combination with nimotuzumab (400 mg weekly, QW) will be determined based on safety and preliminary efficacy evaluation. Phase II will employ Simon’s two-stage minimax design, with planned enrollment of approximately 20 participants. The primary endpoints for Phase Ib are safety profile characterization and RP2D determination; secondary endpoints include objective response rate (ORR), progression-free survival (PFS), overall survival (OS), disease control rate (DCR), and duration of response (DoR). For Phase II, the primary endpoint is ORR, with secondary endpoints comprising PFS, OS, DCR, safety, and DoR.

**Discussion:**

This exploratory clinical trial may yield critical safety/efficacy data supporting novel combination therapy for advanced PDAC. Its findings could advance the application paradigm of dual-target inhibition in pancreatic cancer therapeutics.

**Trial registration:**

This study was registered on ClinicalTrials.gov with NCT06773130.

**Ethics:**

This study protocol has been approved by the Ethics Committee of West China Hospital [2024 (2239)].

## Introduction

Pancreatic tumors are characterized by aggressive invasiveness and high mortality rates. Given their insidious onset, rapid progression, and poor prognosis, they consistently rank among the leading causes of cancer-related mortality (Stoffel et al., 2023). Pancreatic ductal adenocarcinoma (PDAC) constitutes approximately 90% of all pancreatic malignancies (Wolfgang et al., 2013). Due to the absence of effective early diagnostic methods, only 15%–20% of PDAC patients present with resectable localized disease at diagnosis, while the majority are diagnosed at advanced stages, resulting in a 5-year survival rate below 10% (Kamisawa et al., 2016).

Systemic chemotherapy represents the cornerstone treatment for advanced pancreatic cancer (Domagała-Haduch et al., 2024). The 2024 National Comprehensive Cancer Network (NCCN) Clinical Practice Guidelines in Oncology identify AG (gemcitabine + nab-paclitaxel), FOLFIRINOX, and NALIRIFOX as standard first-line regimens for this malignancy (NCCN, 2024). Nevertheless, therapeutic efficacy remains suboptimal, with median overall survival (mOS) under 12 months and median progression-free survival (mPFS) below 8 months (Wainberg et al., 2023). This underscores the imperative to develop innovative therapeutic strategies for advanced disease management.

The epidermal growth factor receptor (EGFR), a transmembrane glycoprotein and member of the ERBB receptor tyrosine kinase family, plays a pivotal role in tumorigenesis by activating Ras/Raf/MEK/ERK, JAK/STAT, and PI3K/AKT signaling pathways (Voldborg et al., 1997). EGFR overexpression is demonstrated in 30%–89% of pancreatic carcinomas (Philip and Lutz, 2015). EGFR-targeted therapies have been employed in pancreatic cancer management for years. Notably, nimotuzumab is approved by China’s NMPA for KRAS wild-type locally advanced or metastatic pancreatic cancer in combination with gemcitabine. The Chinese Society of Clinical Oncology (CSCO) Pancreatic Cancer Guidelines recommend nimotuzumab plus gemcitabine as first-line and beyond therapy for advanced KRAS wild-type PDAC (Duan et al., 2024). However, treatment-refractory recurrent or metastatic pancreatic cancer patients frequently develop chemotherapy intolerance (NCCN, 2024). Consequently, incorporating nimotuzumab-based targeted therapy may represent a novel strategic approach.

The KRAS gene, a key member of the RAS family, orchestrates critical cellular signaling by transducing signals from upstream receptors (including EGFR) to downstream effectors. This activates the RAF-MEK-ERK and PI3K-AKT-mTOR pathways, thereby driving tumor proliferation. KRAS mutations occur in ∼88% of PDAC, with G12D constituting 39.5% of cases (Duan et al., 2024). Consequently, targeting KRAS G12D has emerged as a promising therapeutic strategy. Recent advances in KRAS inhibitor development include G12C-targeting agents (e.g., sotorasib, adagrasib) and G12D inhibitors (e.g., MRTX1133, HRS-4642). Monotherapy with KRAS G12C inhibitors achieves objective response rate (ORR) of 20%–42% in KRAS-mutated PDAC, demonstrating initial clinical efficacy (Nagasaka et al., 2021). Crucially, preclinical studies reveal that EGFR feedback activation mediates resistance to KRAS G12D inhibitors like MRTX1133 (Feng et al., 2023). Co-administration of the EGFR monoclonal antibody cetuximab enhances cellular sensitivity to MRTX1133, inducing tumor regression in KRAS G12D-mutant xenograft models (Hallin et al., 2022). This synergism is clinically validated in the KRYSTAL-1 trial: while adagrasib (KRAS G12C inhibitor) monotherapy yielded 16% ORR in colorectal cancer, combination with cetuximab increased ORR to 46% (Ou et al., 2022; Negrao et al., 2023). These findings demonstrate that combined KRAS/EGFR inhibition enhances therapeutic efficacy. HRS-4642 injection is a long-acting, liposome-formulated KRAS G12D inhibitor with high specificity and potency. It selectively binds to KRAS G12D, suppressing MEK and ERK phosphorylation to exert antitumor effects. Preclinical studies demonstrate that in a KRAS G12D-mutant AsPC-1 human pancreatic cancer xenograft model (nude mice), intravenous administration of HRS-4642 significantly inhibits tumor growth in a dose-dependent manner with favorable tolerability (Zhou et al., 2024).

In conclusion, combined KRAS G12D inhibition and EGFR monoclonal antibody therapy is expected to delay acquired resistance in solid tumors, thereby improving overall prognosis and survival outcomes. This clinical study therefore aims to assess the efficacy and safety of the novel dual-targeting strategy using the KRAS G12D inhibitor HRS-4642 injection plus the EGFR monoclonal antibody nimotuzumab in patients with recurrent/metastatic PDAC harboring KRAS G12D mutations following prior therapy failure.

## Methods

### Study objectives

This single-center, single-arm, open-label phase Ib/II exploratory clinical trial aims to evaluate the preliminary efficacy and safety of the novel KRAS G12D inhibitor HRS-4642 combined with the EGFR monoclonal antibody nimotuzumab in patients with relapsed or metastatic PDAC harboring KRAS G12D mutations.

This two-phase study enrolled patients with recurrent/metastatic PDAC harboring KRAS G12D mutations. Phase Ib evaluated the tolerability and safety profile of HRS-4642 combined with nimotuzumab. Phase II assessed the efficacy and safety of this combination therapy.

#### Phase Ib

• Primary endpoints: Safety, RP2D
• Secondary endpoints: ORR, DCR, PFS, OS, and DoR based on RECIST 1.1,

#### Phase II

• Primary endpoint: ORR
• Secondary endpoints: PFS, OS, DCR, and DoR based on RECIST 1.1, and safety

### Study design

**FIGURE 1:**
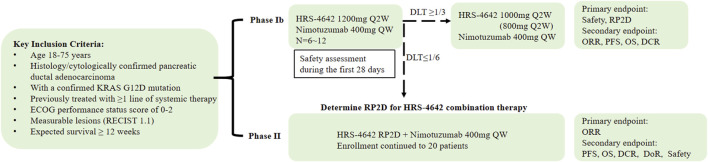
Outline of the study design.

This two-phase study (design schema in Figure 1) comprises:

Phase Ib: Determination of the Recommended Phase II Dose (RP2D) and safety assessment of HRS-4642 plus nimotuzumab. Six participants will initiate combination therapy with intravenous HRS-4642 (1,200 mg Q2W) and nimotuzumab (400 mg QW). Following a 28-day dose-limiting toxicity (DLT) evaluation period:If DLT incidence ≥33.3% (≥2/6 patients), dose reduction (1,000 mg or 800 mg Q2W) or study termination will be implemented. If DLT incidence <16.7% (0/6 patients), the current dose will proceed to Phase II as RP2D.
Phase II: Efficacy evaluation of the combination regimen in 20 patients (including Phase Ib RP2D cohort) using Simon’s two-stage minimax design. Treatment continues until disease progression (PD), unacceptable toxicity, subject/investigator decision to withdraw, or study completion per protocol.

### Eligibility criteria and enrollment

Eligible patients must have: 1) Histologically or cytologically confirmed PDAC with documented KRAS G12D mutation; 2) Disease progression following ≥1 line of standard systemic therapy. Key inclusion criteria include: 1) Age 18–75 years; 2) ECOG performance status 0–2; 3) Life expectancy ≥12 weeks; (4) ≥1 measurable lesion per RECIST v1.1. Full inclusion/exclusion criteria are provided in Table 1.

**TABLE 1:** The key eligible criteria of this trial.

Key inclusion and exclusion criteria	
Inclusion criteria	Exclusion criteria
Aged 18–75 years old (including both end values)	Previous treatment with KRAS inhibitors or targeted EGFR therapy
Pathologically (histologically) or cytologically confirmed pancreatic ductal adenocarcinoma	Known allergy to the investigational drug or any of its components
KRAS G12D mutation	Systemic antitumor therapy including chemotherapy, targeted therapy, and immunotherapy within 28 days before initial administration
Failed first-line or later-line standard-of-care systemic therapy or recurrence or metastasis occurred within 6 months of adjuvant therapy	Patients with prior or co-existing other malignancies, unless complete remission has been achieved at least 2 years prior to screening and no other treatment is required or expected to be required during the study period
At least one measurable tumor lesion (RECIST 1.1)	Accompanied by untreated or active CNS
Eastern Cooperative Oncology Group performance status (ECOG) score 0-2	Have a congenital or acquired immune deficiency
Life expectancy exceeding 12 weeks	Esophageal and gastric varices bleeding caused by portal hypertension occurred within 3 months before first administration
Major organs are functioning normally	Advanced patients who have symptoms, that have spread to the internal organs, and are at risk of developing life-threatening complications in the short term
AE due to previous antitumor therapy must be restored to ≤ Grade 1 (CTCAE v5.0) or the level specified by the exclusion criteria	The presence of gastrointestinal obstruction or signs and symptoms of gastrointestinal obstruction within 6 months before starting treatment, but screening may be performed if surgical treatment has been performed and the obstruction has been completely resolved
Women of childbearing age who have a negative pregnancy test and are not breastfeeding	Acute or chronic pancreatitis with significant clinical significance; Patients at high risk for pancreatitis, such as those with serum amylase and/or lipase concentrations ≥3 ULN
The subjects voluntarily joined the study, and the compliance was good	Participated in a clinical trial of any drug or medical device within 4 weeks prior to initial dosing

CNS, central nervous system metastases; ULN, upper limit of normal.

Following screening completion, baseline assessments will be conducted within 28 days prior to treatment initiation. Eligible patients will undergo comprehensive evaluations including: Demographic and clinical characteristics, tumor/medical history documentation, ECOG performance status (0–2), physical examination with vital signs, laboratory analyses (hematology, biochemistry, coagulation, urinalysis, stool testing, cardiac enzymes), virology screening (HIV/HBV/HCV serology), cardiac assessment (ECG and echocardiography), tumor imaging per RECIST v1.1, quality of life (QoL) questionnaires, pregnancy testing (females) and medication allergy history. Complete assessments detailed in Table 2. Enrollment requires written informed consent obtained, full eligibility confirmation and comprehensive protocol education covering study objectives, procedures, risks/benefits, and patient rights. The consent process complies with Institutional Review Board (IRB)-approved protocols and local regulatory requirements, ensuring patient rights and privacy protection.

**TABLE 2:** Schedule of patient visits and assessments.

Time points Visiting item	Screening period		During treatment								End of treatment/exit	Follow-up period	
	Within 28d before administration	Within 7d before administration	Cycle 1				Cycle 2∼Cycle x						
			D1	D8	D15	D22	D1	D8	D15	D22		Safety follow-up	Survival follow-up
Signed informed consent	√												
Demographic data	√												
Tumor history	√												
Past medical history	√												
Drug allergy history	√												
Combination medication/therapy	√												
Adverse event	√												
Blood routine examination		√		√	√	√	√	√	√	√	√	√	
Blood biochemistry		√		√	√	√	√	√	√	√	√	√	
Urinalysis		√			√		√				√	√	
Stool routine		√	According to clinical indications								√		
Coagulation function		√					√				√		
Virological examination	√		If hepatitis B virus carriers are enrolled, hepatitis B virus nucleic acid load monitoring is required every 3 cycles, and continuous antiviral therapy										
Pregnancy test		√											
Myocardial zymogram		√											
Tumor marker	√						√				√		
Vital sign		√		√	√	√	√	√	√	√	√	√	
Physical examination		√					√				√	√	
ECOG PS		√					√				√	√	
ECG		√			√		√		√		√	√	
echocardiography		√											
HRS-4642			√		√		√		√				
Nimotuzumab			√	√	√	√	√	√	√	√			
Imaging examination	√		Every 8 weeks for the first 48 weeks; After 48 weeks, every 12 weeks								√		

### Intervention

Patients will receive intravenous HRS-4642 every 2 weeks (Q2W) plus intravenous nimotuzumab 400 mg weekly (QW) in 28-day cycles. Treatment continues until: treatment completion, unacceptable toxicity, disease progression (RECIST v1.1), withdrawal of consent, loss to follow-up, death, study termination by sponsor, whichever occurs first.

### Efficacy assessment

Tumor response was assessed per RECIST v1.1 using contrast-enhanced CT (preferred) or magnetic resonance imaging (MRI). Imaging modality selection was at investigator’s discretion; however, consistent equipment and acquisition protocols were mandated throughout the study. Assessments occurred every 8 weeks (±7 days) for the initial 48 weeks post-first dose, transitioning to every 12 weeks (±7 days) thereafter. First-time PR/CR required confirmation ≥4 weeks later. The ±7-day imaging window was protocol-permitted and independent of treatment interruptions. Participants discontinuing treatment without progression should continue scheduled imaging until: radiologic progression, death, loss to follow-up, consent withdrawal, new antitumor therapy initiation, investigator-determined study termination, whichever occurs first.

### Safety assessment

Safety assessments must precede each treatment cycle, encompassing: adverse events (AEs) and serious AEs (SAEs); vital signs and physical examinations; 12-lead ECGs; laboratory tests (including pregnancy testing); and concomitant medications, with visit-specific procedures detailed in Table 2. Participants require vigilant post-dose monitoring throughout the trial, with prompt AE management to ensure subject safety. According to the established protocol: 1) The study drug should be discontinued in the event of hypersensitivity reactions of grade 3 or higher; 2) HRS-4642 must be permanently terminated in cases of grade 4 drug-related toxicity; 3) Treatment discontinuation is at the investigator’s discretion for any additional safety concerns.

After the resolution of adverse events (AEs), it is imperative to meticulously document the type and symptoms of the event, the timing of onset and resolution, the severity according to the National Cancer Institute’s Common Terminology Criteria for Adverse Events (NCI CTCAE) version 5.0, the management strategies employed, and the outcomes. This documentation is essential for making informed decisions regarding the continuation of the trial. All AEs, including those occurring during the screening phase, will be assessed for type, incidence, CTCAE grade, time to onset and resolution, classification as serious adverse events (SAEs), causality, and clinical outcomes. SAEs are defined as events that result in death, are life-threatening, require hospitalization or prolongation of existing hospitalization, lead to persistent or significant disability, or result in congenital anomalies.

### Sample size design

The sample size for the Phase Ib study (n = 6–12) is determined based on observations of toxicity rather than statistical calculations. For the evaluation of efficacy in Phase II, Simon’s two-stage minimax design will be employed. Previous research indicates an ORR of 20%–42% for KRAS G12C inhibitor monotherapy in patients with pancreatic cancer who have undergone at least two lines of prior therapy (Strickler et al., 2023; Bekaii-Saab et al., 2023; ASCO GI oral 604, 2024; ASCO Oral Abstract, 2024), compared to a 7% ORR for the combination of gemcitabine and nimotuzumab (an EGFR inhibitor) (Qin et al., 2023). The statistical hypotheses are as follows: null hypothesis (H0): ORR ≤7%; alternative hypothesis (H1): ORR ≥27%, with a one-sided significance level (α) of 0.05 and a power of at least 80%. This design necessitates 20 evaluable subjects. In Stage 1, 9 participants will be enrolled, and progression to Stage 2 will require at least one confirmed CR/PR. If this criterion is met, Stage 2 will enroll up to a total of 20 subjects (an additional 11 patients), with the study deemed successful if there are at least four CR/PR events in the entire cohort.

### Data analysis

Analyses will be performed according to the ITT principle, patients with final missing outcomes will be assumed to be treatment failures. Analysis groups were classified into full analysis set (FAS), Efficacy Evaluable Analysis set (EES), and safety set (SS).

- The FAS comprises a dataset derived from all participants who administered the study drug at least once in accordance with the protocol.
- The EES is a subset of the FAS, consisting of individuals who received the investigational drug without committing any significant violations of the investigational protocol and had at least one efficacy evaluation result.
- The SS includes individuals who received at least one injection of the investigational drug and provided informed consent.

ORR and DCR were summarized using descriptive statistical methods, and 95% confidence intervals were calculated (Clopper-Pearson method). For OS, PFS, and DoR, the Kaplan-Meier method was used to plot survival curves, and the median time and corresponding 95% confidence interval were estimated (Brookmeyer-Crowley method based on log-log transformation). The incidence of AEs, treatment-related AEs, SAEs, and AEs leading to study withdrawal will be summarized.

All the statistical analyses will be conducted using SPSS version 22 or above.

## Discussion

Given the suboptimal survival rates linked to existing treatment protocols and clinical trials for individuals with advanced PDAC, there is an urgent need to develop more effective therapeutic strategies to enhance patient survival. This study proposes a design to assess the safety and efficacy of dual-targeted therapies, specifically the KRAS G12D inhibitor HRS-4642 and the EGFR-targeting monoclonal antibody nimotuzumab, in patients with recurrent or metastatic PDAC who have not responded to systemic treatments and possess KRAS G12D mutations.

Previous studies have indicated that the concomitant use of EGFR inhibitors and KRAS inhibitors can yield clinical benefits in the treatment of non-small cell lung cancer (NSCLC) and colorectal cancer (Gregorc et al., 2024; Zhou et al., 2023; Yaeger et al., 2023). In this study, nimotuzumab, an EGFR inhibitor approved for pancreatic cancer therapy, will be utilized. Additionally, HRS-4642, a selective KRAS G12D inhibitor formulated as a liposomal injection, will be employed. This liposomal formulation is intended to improve the drug’s distribution and metabolism within the body and tumor, thereby enhancing efficacy while reducing toxicity. HRS-4642 is currently being assessed in a Phase I clinical trial (HRS-4642-I-101) involving patients with advanced solid tumors harboring the KRAS G12D mutation (Zhou et al., 2023). Although the combination of HRS-4642 and nimotuzumab has not yet been investigated in human studies, the potential for unexpected risks or toxicities associated with this combination remains uncertain. Nonetheless, based on the known toxicity profiles of HRS-4642, nimotuzumab, and similar agents, it is anticipated that the combined use of these two drugs will demonstrate a lower toxicity profile. However, there is a potential for an increased risk of aminotransferase elevation and anemia.

This study seeks to furnish compelling evidence for the clinical implementation of dual-targeted therapies, namely, the KRAS G12D inhibitor HRS-4642 and the EGFR-targeting monoclonal antibody nimotuzumab, in patients with recurrent or metastatic PDAC harboring KRAS G12D mutations and exhibiting resistance to systemic therapy.

## Data Availability

The original contributions presented in the study are included in the article/supplementary material, further inquiries can be directed to the corresponding author.
